# Drastic Multiorgan Dysfunction Due to Severe Leukostasis: A Case Report

**DOI:** 10.7759/cureus.31518

**Published:** 2022-11-15

**Authors:** Akinori Tada, Yasuhiro Kikuchi, Kazuyuki Murase, Kohichi Takada, Akira Takasawa

**Affiliations:** 1 Department of Pathology, Sapporo Medical University School of Medicine, Sapporo, JPN; 2 Department of Medical Oncology, Sapporo Medical University School of Medicine, Sapporo, JPN

**Keywords:** end-stage, multiple organ dysfunction, multiple organ infarction, acute myeloid leukemia, hyperleukocytosis, leukostasis

## Abstract

Leukostasis is a life-threatening complication that causes vascular occlusion leading to organ damage in leukemia patients. Organs with impairment due to leukostasis are usually the lungs and kidneys, but other organs may also be damaged. We experienced an autopsy case of severe infarction in multiple organs including the spleen probably due to leukostasis in a patient with acute myeloid leukemia.

## Introduction

Acute myeloid leukemia (AML) is a hematologic malignancy characterized by the rapid growth of abnormal myeloid precursors [1]. Most AML patients have immunosuppression and bleeding tendencies because leukemic cells occupy the bone marrow and prevent normal hematopoiesis. As a result, many patients with AML die from severe infection and hemorrhage. Multiple organ failure, a clinical syndrome due to the failure of several organs that is fatal without intervention [2], is also a possible cause of death in AML patients [3], but there have been surprisingly few detailed histopathologic reports on such cases [4]. Herein, we describe multiple organ dysfunction syndrome due to multiple organ infarctions based on an autopsy case we experienced.

## Case presentation

Clinical course

A 69-year-old Japanese man under follow-up of aplastic anemia was admitted to our hospital due to an elevated white blood cell count (WBCC) and abscess in the gluteal region. To assess the detailed condition of the patient, a bone marrow biopsy, blood tests, and a chromosome examination were performed. The bone marrow biopsy showed an abnormally high myeloblast ratio of 22% and dysplasia in three types of blood cell lineages. The blood tests showed a low hemoglobin concentration of 9.0 g/dL, a low platelet count of 58 x 10^3^/µL, and a low neutrophil count of 208/µL. The chromosome examination revealed that the tumor cells had i(17)(q10) chromosome abnormality. Taking the results together, the patient was diagnosed with high-risk myelodysplastic syndrome (MDS) based on FAB (French-American-British) classification. Azacitidine therapy was initiated for the treatment of high-risk MDS. After nine months of azacitidine administration, the ratio of blast cells in bone marrow decreased to 11% from 22%, but the ratio increased to 22% six months later. Therefore, the patient was diagnosed with progression to AML. After a thorough and careful discussion with the patient, it was decided not to provide further aggressive treatment. Afterward, the patient’s condition gradually deteriorated, and one month after the diagnosis of AML, he died due to ventricular tachycardia and ventricular fibrillation. One day before his death, a blood test showed a WBCC of 392,700/µl and a ratio of blast cells of 8.8% in peripheral blood. Other blood counts and results of biochemical tests are shown in Table 1.

**Table 1:** Laboratory values one day before the patient’s death

Test	Values	Reference range
Red blood cells	2.2 x 10^6^/μL	4.3 x 10^6^-5.7 x 10^6^ /μL
Hemoglobin	5.9 g/dL	13.4-17.6 g/dL
Hematocrit	16.4%	39.6-52.0%
Platelets	55 x 10^3^/μL	127 x 10^3^-356 x 10^3^ /μL
White blood cells	392.7 x 10^3^/μL	3.9 x 10^3^-9.8 x 10^3^ /μL
Absolute neutrophil count	0.7 x 10^3^/μL	-
Absolute lymphocyte count	0.3 x 10^3^/μL	-
Absolute monocyte count	130 x 10^3^/μL	-
Absolute eosinophil count	3.9 x 10^3^/μL	-
Absolute basophil count	0 x 10^3^/μL	-
Blastoid	8.8%	-
Pro-Myelocyte	4.0%	-
Myelocyte	45.8%	-
Meta-Myelocyte	5.2%	-
Total protein	4.5 g/dL	6.5-8.0 g/dL
Alb	3.1 g/dL	3.7-5.2 g/dL
Total bilirubin	0.8 mg/dL	0.2-1.2 mg/dL
Direct bilirubin	0.4 mg/dL	0.0-0.3 mg/dL
Creatinine kinase	9 U/L	71-220 U/L
Aspartate aminotransferase	35 U/L	11-39 U/L
Alanine aminotransferase	76 U/L	5-40 U/L
Lactate dehydrogenase	2232 U/L	119/229 U/L
Alkaline phosphatase	443 U/L	110-370 U/L
Creatinine	1.51 mg/dL	0.55-1.04 mg/dL
Urea nitrogen	67 mg/dL	6-20 mg/dL
C reactive protein	0.44 mg/dL	0.00-0.30 mg/dL

An autopsy was performed to determine the patient’s detailed condition in the terminal stage.

Autopsy findings

**Figure 1:**
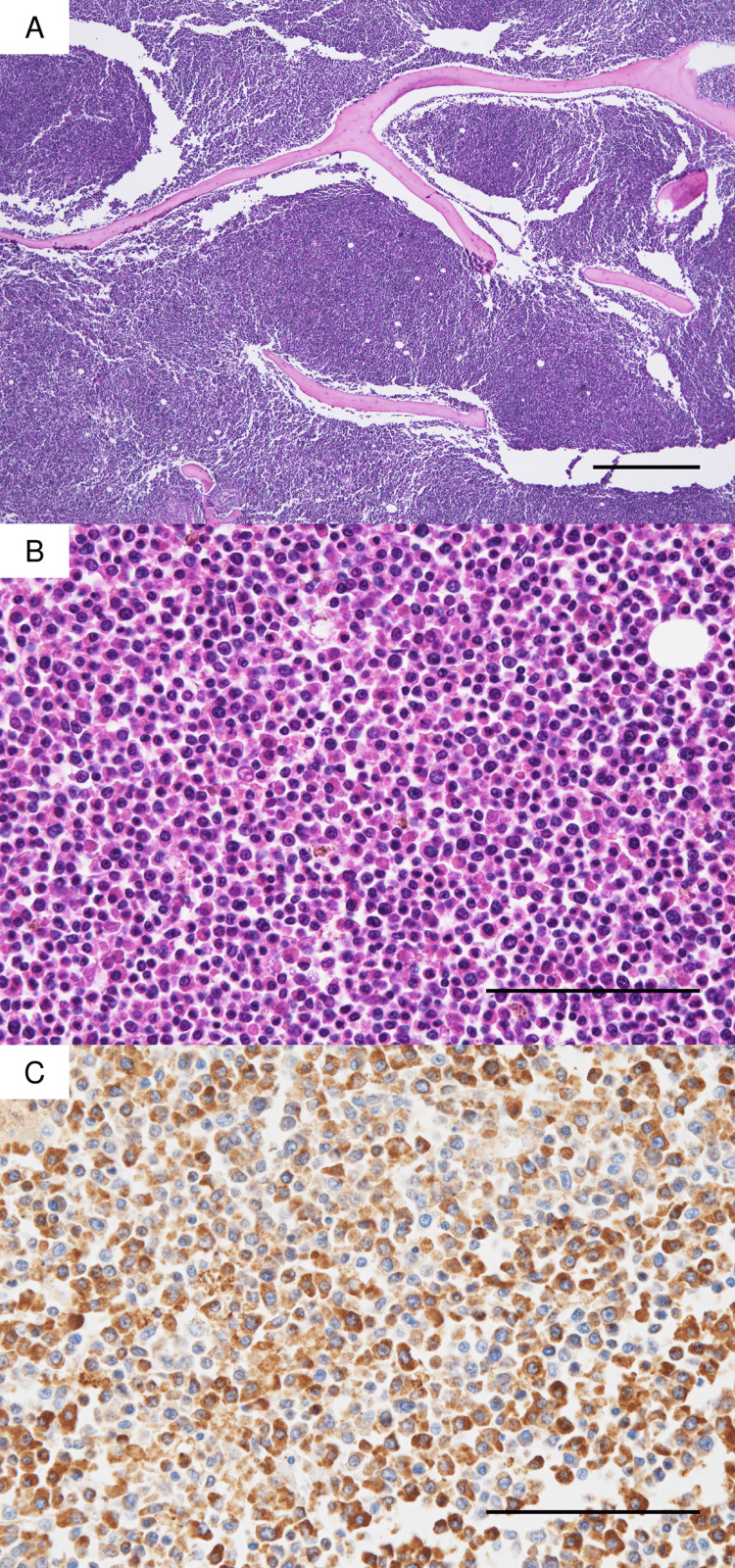
Microscopic findings of bone marrow. (A) Low power view of bone marrow. H&E staining 4x, Bar: 500 μm (B) High power view of bone marrow. H&E staining 4x, Bar: 50 μm (C) High power view of bone marrow. MPO staining 40x, Bar: 50 μm

On external examination, there were no palpable superficial lymph nodes or bleeding tendencies (e.g., subcutaneous hemorrhage, purpura). Microscopically, the bone marrow cellularity showed almost 100% (Figure 1A). At higher magnification, oval tumor cells were diffusely proliferating, with highly atypical nuclei and coarse chromatin (Figure 1B). Most of the bone marrow cells were composed of blast cells with MPO+, but a few non-blast cells with MPO- were also observed (Figure 1C). Immunohistochemically, the leukemic cells showed MPO+, TdT-, CD34-, CD68-, c-kit-, and p53-, and infiltration of such leukemic cells into systemic organs was observed.

**Figure 2:**
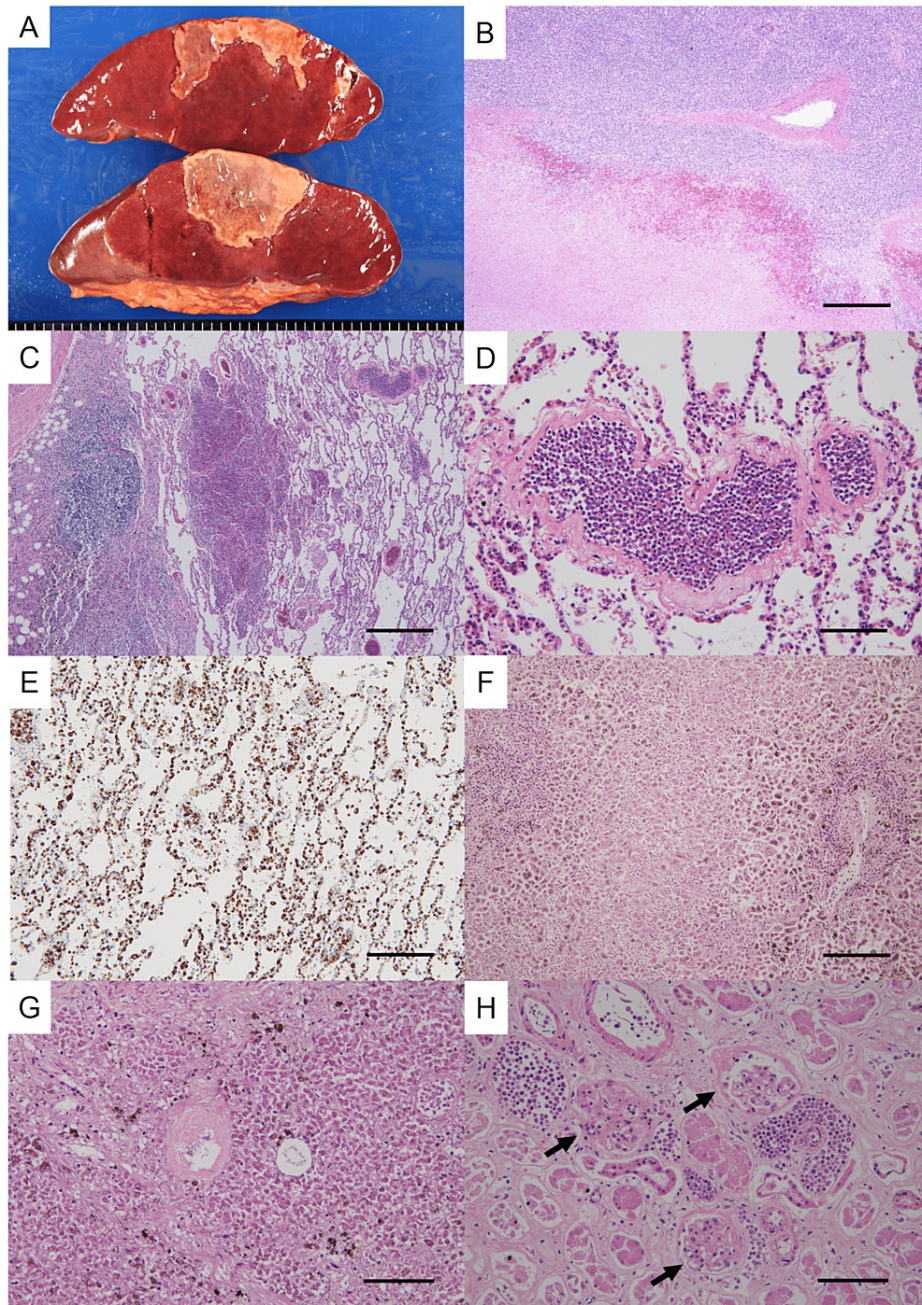
Macroscopic and microscopic findings. (A) Necrotic area on the cut surface of the spleen. (B) Necrotic tissue of the spleen. H&E staining 4x, Bar: 500 μm (C) Leukemic infiltration and hemorrhage in the lungs. H&E staining 4x, Bar: 500 μm (D) A blood vessel filled with leukemic cells in the lungs. H&E staining 20x, Bar: 100 μm (E) The leukemic cells were arranged along the alveolar walls. MPO staining 10x, Bar: 200 μm (F) Extensive coagulative necrosis in the liver. H&E staining 10x, Bar: 200 μm (G) Coagulative necrosis and leukemic infiltration of the pancreas. H&E staining 20x, Bar: 100 μm (H) Coagulative necrosis of renal glomeruli (arrows) and leukemic cell infiltration. H&E staining 20x, Bar: 100 μm

The spleen was markedly heavy (weight, 750 grams; reference value, 139 grams [5]). Macroscopically, fibrinoid deposits were found on the external surface, and a well-circumscribed yellowish-brown lesion was observed on the cut surface (Figure 2A). Microscopically, the lesions were occupied with diffuse coagulative necrosis (Figure 2B), and leukemic cells were observed in an extensive area of splenic tissue. In the lungs, there were no abnormal macroscopic findings on the external and cut surfaces, and the weight of the right lung was increased (right lung weight, 644 grams; reference value, 445 grams and left lung weight, 430 grams; reference value, 395 grams [5]. Histological findings showed the destruction of alveolar structures and focal alveolar hemorrhage in the whole lung field with scattered leukemic cells without any acute inflammatory cell infiltration, fibrosis, or mass of microorganisms (Figure 2C). Leukemic cells filled the capillaries of the alveolar walls, as confirmed by MPO immunostaining (Figures 2D, 2E). The liver was markedly increased in weight (2140 grams; reference value, 1561 grams [5]. The liver appeared to be congested in color, macroscopically, without space-occupying lesions. On histological examination, there was extensive necrosis of hepatocytes with bile deposits mainly in the centrilobular region (zone 3), and blast cells mainly infiltrated in the hepatic sinusoids and vessels of Glisson’s capsules (Figure 2F). In the pancreas, microscopic findings showed leukocyte infiltration and fat necrosis in the peripancreatic adipose tissue and expanded coagulative necrosis in the pancreatic parenchyma (Figure 2G). In the kidneys (right kidney weight, 110 grams; reference value, 129 grams and left kidney weight, 130 grams; reference value, 137 grams [5], coagulative necrosis of the proximal and distal tubules was also observed (Figure 2H), and leukemic cells infiltrated mainly in the glomeruli and perirenal adipose tissue. In the heart (weight, 410 grams; reference value, 308 grams [6], the blood vessels in the myocardium were filled with blast cells, but no infarct or inflammatory cell infiltration was observed in the myocardium. In addition, the pericardium was thickened and had inflammatory cell infiltration, and these findings were suggestive of pericarditis.

Considering the findings of expanded alveolar hemorrhage and coagulative necrosis in the parenchymal organs on autopsy, it was expected that the patient was in a state of multiorgan failure caused by leukostasis.

## Discussion

In this case, the patient progressed from MDS to AML, and WBCC was continuously elevated until the patient died. Autopsy also revealed leukemic bone marrow and alveolar hemorrhage, coagulative necrosis of parenchymal organs, and occlusion of blood vessels by leukemic cells. Based on these clinical courses and autopsy findings, it was thought that abnormally proliferating blasts occluded the blood vessels of systemic organs, resulting in a state of multiple organ dysfunction syndrome. The patient’s condition is understood to be leukostasis.

Leukostasis is a severe complication associated with hyperleukocytosis, which is classically defined as a WBCC of 100,000/µl or more [7]. The frequencies of hyperleukocytosis and leukostasis vary depending on the subtype of leukemia, and leukostasis is rarer than hyperleukocytosis in any type of leukemia [8]. The possible pathogenesis of leukostasis includes an extreme increase in WBCC [9], blood hyperviscosity [7,9,10], and abnormal adhesion between the endothelium and blast cells [7,9,11]. Leukostasis usually impairs microcirculation [11,12], and the central nervous system and lungs are vulnerable to leukostasis [7,13]. In this case, pulmonary congestion and renal dysfunction were observed in the terminal stage, and these manifestations were consistent with organ damage due to leukostasis.

In the present case, the autopsy findings revealed alveolar hemorrhage and coagulative necrosis in the parenchymal organs, and the blood vessels were filled with blast cells, suggesting that the patient was in a state of multiple organ dysfunction due to leukostasis. Severe leukostasis in the present case could have been triggered by the extremely high WBCC of 392,700/µl. Therefore, control of WBCC to prevent leukostasis might contribute to the reduction of organ damage in patients with end-stage AML. For this purpose, anticancer agents with few adverse effects such as hydroxyurea might be selectable options. A unique aspect of this case is the extensive infarction in multiple organs, especially the spleen. Multiple organ dysfunction caused by leukostasis as in the present case has rarely been reported [14], whereas there have been several reports of leukostasis in a single organ such as the lung, kidney, or brain [15,16]. Moreover, the extensive necrosis of the spleen, in this case, suggests obstruction of a large blood vessel in the spleen. Since the spleen receives blood supply only from the splenic artery, the splenic tissue is prone to ischemia and consequently to infarction if the blood supply of the splenic artery is disrupted [17]. In the present case, it is thought that the blood flow obstruction caused by leukostasis resulted in an extensive infarction of the spleen. Although relatively large vessels such as coronary arteries could be occluded by leukostasis [18,19], there has been no previous report of leukostasis with embolism of relatively large vessels and extensive necrosis in the spleen. To the best of our knowledge, this is the first report of splenic necrosis that appears to be caused by occlusion of large blood vessels in the spleen, described as leukostasis.

## Conclusions

This autopsy case demonstrates the significantly adverse impact of leukostasis on patients with end-stage AML. Multiorgan dysfunction due to leukostasis has rarely been reported, although a WBCC over 100,000/µL seems to be relatively common in end-stage AML. This dissociation may reflect the fact that refraining from aggressive treatment and examination of patients with end-stage AML could mask these conditions of leukostasis. Through this case study, we have demonstrated the unrecognized severity of leukostasis in patients with end-stage AML and the potential benefit of WBCC control for the prolonged prognosis of such patients. Early detection and intervention for leukostasis should be considered when treating AML patients if clinical manifestations suggest leukostasis.
